# Triple-hit explanation for the worse prognosis of pediatric acute lymphoblastic leukemia among Mexican and Hispanic children

**DOI:** 10.3389/fonc.2022.1072811

**Published:** 2022-11-30

**Authors:** Roberto Rivera-Luna, Patricia Perez-Vera, Cesar Galvan-Diaz, Liliana Velasco-Hidalgo, Alberto Olaya-Vargas, Rocio Cardenas-Cardos, Marco Aguilar-Ortiz, Jesus Ponce-Cruz

**Affiliations:** ^1^ Department of Oncology, National Institute of Pediatrics, Mexico City, Mexico; ^2^ Pediatric Oncology Unit, ABC Medical Center, Mexico City, Mexico; ^3^ Laboratory of Genetics and Cancer, National Institute of Pediatrics, Mexico City, Mexico; ^4^ Progenitor and Hematopoietic Cell Transplant Unit, National Institute of Pediatrics, Mexico City, Mexico

**Keywords:** acute lymphoblastic leukemia, pediatric, Hispanic, Mexican, survival gap

## Abstract

Acute lymphoblastic leukemia (ALL) is the most common malignancy among Mexican and Hispanic children and the first cause of death by disease in Mexico. We propose a “triple-hit” explanation for the survival gap affecting this population. The first hit can be attributed to epidemiology and social, cultural, and economic burdens. The second hit refers to cancer biology, with a high incidence of unfavorable genetic characteristics associated with an unfavorable response to treatment and, subsequently, poor survival. Finally, the third hit relates to sub-optimal treatment and support. Society and culture, leukemia biology, and treatment approach limitations are key factors that should not be seen apart and must be considered comprehensively in any strategy to improve the prognosis of Mexican and Hispanic children with ALL.

## Epidemiological considerations in pediatric ALL

Mexico is among the top five countries that send emigrants to the United States of America (USA). For legal and nonlegal Mexican immigrants living in the USA, the mortality attributed to cancer is comparable to Mexicans living in Mexico (1). Childhood cancer is aggravated by the increasing poverty levels that affect Mexican and Hispanic (a person with ancestry from a Spanish-speaking territory or country) communities in the USA.

Recently, close to 12 million immigrants to the USA from Mexico have left (INEGI) (2). In total, 74% are illegal, most of whom will eventually become legal aliens. According to the United States Census Bureau (3), in 2020, there will be 62 million Hispanic citizens; some 20 million are under 19 years, and this number does not include undocumented immigrants. With this background, the US health system is under pressure, particularly when facing children with cancer (4). Worldwide, in **high**-income countries (HIC), the cost of diagnosing and treating children and young adults with cancer is estimated at $28,000/year (5). Despite universal health care in some countries, for low-middle income countries (LMIC), it has become a burden for parents, their families, and their country (5, 6). A small percentage of these children return to Mexico for medical assistance when they develop cancer. However, those who remain in the USA face adversity, despite the different financial approaches to lower the cost of medical care (5, 7).

Mexico’s public health policies have changed since 2019 by altering a previous national childhood cancer program (6). Nowadays, Mexico has a shortage of supplies, personnel, and facilities to treat children with ALL. Mexico has only 60 accredited hospitals, most of them general hospitals with a pediatric oncology department. Around 380 board-certified pediatric oncologists work hard every day to cover the needs of the children in the country. The national shortage of chemotherapy, the overwhelming mortality, and the financial cost of the coronavirus 2019 disease pandemic have strongly affected the Mexican Public Health services, causing childhood cancer care to fail. This includes the socialized medical institutions and the noninsured patients under the welfare system, which covers over 50% of all children with cancer in Mexico (6, 8).

Given the current data from the USA, around 50% of Hispanic children with cancer have leukemia; these children have a high incidence of biological characteristics associated with a poor response to treatment and, consequently, high mortality risk. This is comparable to data from Mexico, where ALL accounts for 50% of all types of cancer in children aged 0-18 years, with the highest incidence among children aged 1–4 years (8–12).

In 2017, we reported that the mortality rate of Mexican children with cancer has not diminished; the adjusted rate was 5.3/100,000/year, with a peak of 8.6/100,000/year in the 15–18 year-old group (xref>/xref>). In the USA, among Hispanics, in those aged 0–14 years, the mortality rate was 2.2/100,000 from 2006 to 2010, and in those aged 15-19 years, it was 3.2/100,000/year, which is the highest rate by race/ethnicity (Hispanics) in the USA, and mainly accounted for by patient with ALL (1, 12, 13).

### First hit: Culture and society

The described findings define pediatric acute lymphoblastic leukemia (ped-ALL) as a public health matter in Mexico.

Recent studies suggest that, despite the country of residency or immigration status, children residing in Hispanic enclaves in HIC experience poorer overall survival, finding that society and culture represented by living in areas with high concentrations of Hispanic immigrants, ethnic-specific businesses, and language isolation (that often implies a socioeconomic deprivation) are determinants of a lower overall survival (13).

Hispanic children in the USA have a higher incidence of ALL than Caucasian and Black children. Census data show that legal Mexican first-generation and the Mexican American population account for 62 million people in the USA (14). Therefore, according to the census of Mexican Americans and the recent legal and illegal immigrants, approximately 74 million Hispanics live in the USA, which makes up 22% of the total population (4, 6). There are 20 million Hispanic children of Mexican descent, according to Pew Research Center findings in 2019. The research results from this center outlined that 27% of this Hispanic Mexican population are under the limits of poverty, and 34% are without any type of Health Insurance (12). Additionally, low income favors a poor prognosis in US children regardless of race (15).

### Second hit: Unraveling the enigma of ALL biology among Hispanics

Initial theories attributing this burden exclusively to socioeconomic factors have been broadly studied, resulting in clinical model proposals implemented in LMIC (16, 17). However, Hispanic children are between 1.2 and 1.75 times more likely to develop ALL and have a 40% higher death rate than their counterparts even after correcting for socioeconomic factors (17–20). Since the outcome for Hispanics with Ped-ALL is worst despite the country of treatment or socioeconomic condition, interest in a biological explanation has arisen over the past ten years (21). Important differences in the genetic background of Hispanic patients have been revealed (22, 23).

When analyzing leukemia in Hispanics, some genetic results are conditioned by the ethnic composition of the study population (24). For example, a California study found that acute promyelocytic leukemia (APL) is more commonly diagnosed in Hispanic adults, and non-APL acute myeloid leukemia (AML) appears less common in Hispanics (25). In contrast, a study made in Florida showed that AML was more common in Hispanics, regardless of subtype (24). This discrepancy may be associated with ethnic background; in California, 84% of the Hispanic population is from Mexican origin; in Florida, this is only 23.6%, demonstrating the heterogeneity among the Hispanic population (24). These observations revealed that the genetic background of Mexicans is involved in the biology and presentation of leukemia.

Since T cell ALL is less common among Hispanic patients, differences are mainly attributed to B cell precursors (pre-B) ALL, which is highly incident and presents individual differences in genetic characteristics (24). Genetics studies of patients diagnosed at our Institution in Mexico City revealed discrepancies in the frequencies of the classical abnormalities in this subtype. **Table 1** demonstrates the incidence of recurrent translocations, ploidy levels, and frequent abnormalities observed in patients with pre-B ALL without recurrent translocations (B-other) in Mexican, Hispanic, and non-Hispanic patients from Los Angeles, California. The most relevant differences in Mexican patients are: the lower incidence of t (12, 21)(*ETV6::RUNX1*), similar to Hispanics (22, 26). The t (1, 19)(*TCF3::PBX1*) is slightly more frequent in Mexicans than in Hispanics and non-Hispanics (22, 27); abnormalities in *CRLF2* are particularly frequent in Mexicans, with a similar incidence of *IGH::CRLF2* compared to Hispanics, and *P2RY8::CRLF2* is present with a higher frequency, all patients with these rearrangements presented CRLF2 overexpression (28–30); the iAMP21 is also frequent (10.1% in B-Others) (26, 29). This means that in Mexicans, *ETV6-RUNX1*, associated with standard prognosis, is reduced, and rearrangements of *CRLF2* and *iAMP21*, which confer high risk, are widely represented among Mexican patients. Most of these findings have been replicated and confirmed by other groups in Mexico (**Table 1**) (31, 32), although they must be compared to data from other regions in our country.

**Table 1:** Comparative incidence of recurrent genetic abnormalities in mexicans, hispanics and non-hispanics children diagnosed with pre-b all.

	Mexicans National Institute of Pediatrics (26, 27, 29,30)	Mexicans Other Institutions (31,32)	Hispanics (22,30)	Non- Hispanics (22, 30)
Translocation Profiles (%)				
t(12;21) ETV6::RUNX1	8.4	10.5-14.9	12.6	24
t(1;19) TCF3::PBXI	7.2	5.8-7.7	–	–
t(17;19) TCF3::HLF	0.6	–	–	–
19p13 TCF3	–	–	4.6	4.7
t(4;11) KMT2A::AFFI	1.4	2.8-4.2	–	–
11q23 KMT2A	2.2	–	2	2.9
t(9;22) BCR::ABLI	3.7	3.3-7.3	1.3	0.7
Ploidy Level (%)				
Hyperdiploid				
>50	25.3	–	41.1	36.6
47-50	16	–	13.9	18.6
Hypodiploid				
41-45	8.6	–	6	8.5
B-Other Abnormalities (%)				
CRLF2 Rearrangements P2RY8-CRLF2	20.5	–	5.5	8
IGH-CRLF2	9.6	–	12	2.7
iAMP21	10.1	–	–	–

Likewise, specific polymorphisms associated with the risk of developing pre-B ped-ALL and lower survival rates have been detected more frequently in Hispanic patients (23). In a cohort of Mexican patients with ped-ALL, we analyzed seven polymorphisms in the transcription factor *ARID5B* gene, including rs10821936. We found that the frequency of all risk alleles was higher in our patients than in Hispanics with ped-ALL residing in the USA (23, 33).

The polymorphisms of genes involved in the metabolism of chemotherapeutic agents are another crucial biologic characteristic that affects the prognosis of ALL and differs among populations. Thiopurine S-methyltransferase (TPMT) participates in the toxicity and therapeutic efficiency of thiopurine drugs such as mercaptopurine, thioguanine, and azathioprine. Patients with low or undetectable levels of TPMT activity have better outcomes. However, such patients may be at higher risk of developing therapy-related toxicities such as myelosuppression and infection (34). In comparison, patients with high TPMT activity present a reduced clinical response to these agents and are prone to relapse (35–37). Based on this, it is possible to adjust the dose of purine analogs according to TPMT activity (34). The frequency of 12 *TPMT* polymorphisms was analyzed in 1270 Mestizos and 20 Mexican Natives and compared to Mexicans of Los Angeles, California (MXLA). Significant differences in allele frequency were detected; the key variant for thiopurine dosing TPMT*3B rs1800460 was two-fold more frequent in Mestizos compared to Natives and 10% higher than in *MXLA* (38). The same study also analyzed the polymorphism *NUD2T*15*3 rs116855232. It has clinical usage since reduces or abolishes the enzyme activity, diminishing tolerance to thiopurines. The *NUDT15* polymorphism was observed with two-fold and 1.3-fold higher allele frequency in Natives and Mestizos compared to Caucasians, respectively. Considering these results, polymorphism analysis of *TPMT* and *NUDT15* must be performed in Mexican children with ALL to prescribe thiopurines (38).

Ped-ALL in Hispanics, particularly Mexicans, differs from other populations towards higher risk. As described (**Table 1**), well-known genetic risk abnormalities are frequently augmented, and good prognosis biomarkers are diminished. Added to the molecular/pharmacogenetic and social factors described the response to treatment contributes to the outcome gap for these patients.

### Triple hit: Limited treatment strategies for Hispanics with ALL

Collaborative work has been crucial for the progress of oncology; cooperative efforts have allowed previously incurable diseases to now have cure rates that exceed 90% in some diseases, breaking a paradigm that associates cancer with a death sentence. These collaborations are based on a structured model of clinical care, which, through the standardization of criteria, structured registries, and clinical care protocols, allows for the continuous evaluation of results and favors the proposal of modifications that translate into less morbidity and more survival for patients optimizing the use of resources (39).

The need to replicate these collaborative models in the regions that serve the Hispanic population (Latin America) is evident; however, despite the linguistic similarity, the social, cultural, and geographical disparity, and the limited resources make this challenge a titanic mission. Global medicine efforts have failed to match the cancer treatment outcomes between LMIC and HIC. Particularly for the Hispanic LMIC, this failure may find an explanation in the ped-ALL biology, which is an adverse factor to the already serious social, economic, and health access issues of this mainly Hispanic population countries (40).

The patients in Mexico face an important challenge, i.e., increased rates of relapse, treatment-related toxicity, and treatment abandonment. Around 7–15% of patients die during the induction phase of the treatment, over 25% of patients with ped-ALL die from treatment toxicity, mainly due to infections, around 25–35% of patients relapse, and the abandonment rate may reach up to 50% in some areas of the country (6, 19, 41).

Limited access to adequate pediatric intensive care, geographical and social barriers for well-timed detection and attention of complications for patients in treatment favor the risk of death during treatment for patients, not only in LMIC like Mexico but also for patients in HIC living in Hispanic enclaves (13, 42, 43).

Recent efforts by both local actors in LMICs and global health initiatives led by HIC institutions are aimed at lowering the survival gap by improving supporting care and applying intensified risk-based treatment protocols with appropriate patient education and treatment response assessment on follow-up, considering a comprehensive approach to society, leukemia biology, and treatment based of risk biologic factors (43, 44).

## Discussion

As overall survival for pediatric patients with ALL in HIC has improved over the past 60 years, marked racial disparities are becoming more and more obvious, particularly to the detriment of the Hispanic children population. Additionally, the prognosis for Hispanic children with ALL in LMIC remains practically unchanged, despite nationwide efforts and the current biologic knowledge of this disease. In recent years in the USA, it has been outlined the rather poor outcome for Hispanic children (15).

Another worrisome fact is the continuous rise in the incidence of ALL in Mexico and the USA. Given this, we should work around the co-factors where this population lives, including not only the biology in each patient, but the environment, the type of food following ancestral traditions which continues up to the present time, the exposure to toxic elements close to the household, and many other factors close to these children (45).

Relapse, treatment-related mortality, lack of well-timed and adequate support treatment, and treatment abandonment are problems for Hispanics and Mexicans treated in LMICs and Hispanic enclaves in HICs. While a reduced-intensity treatment may be an obvious approach to solving these problems, the well-underlined biological features of ped-ALL in Hispanics limit the benefit of a low-intensity therapy. These factors may explain the limited success of existing treatment approaches (46). We should seek risk-adapted treatment, but this requires an exhaustive study of the biological features of ALL at the time of diagnosis, and adequate high sensitivity detection of residual disease burden over the treatment to appropriately adapt treatment intensity. Still, in most LMICs, this is not always possible.

The current socioeconomic status, race, and biological changes present among Mexican children and Hispanics in the USA are factors to be considered in designing a multidisciplinary approach to improving the outcome of this population (1, 8, 12, 15, 46).

There is no question about ALL among these populations and the prevalence of several biologic factors that so far have no explanation, including incidence, race, and biological markers. In any event, there is a need for further analysis of molecular epidemiology. Ongoing study in this area is re to elucidate all these findings.

The ideal clinical care model for ped-ALL in LMIC has not yet been described. Cardinal points have been considered, such as universal healthcare access, continuing medical education for early diagnosis, cancer screening access for children’s main malignancies, national cancer registries, an effective approach for the complications during cancer treatment, access, and improving the quality of hospice and palliative care for patients requiring end of life support. Nevertheless, this should be considered the minimum requirement and not the optional treatment for ped-ALL. Local investment in healthcare quality models and basic, clinical, and cancer-biology research should also be prioritized and not be seen as exclusive to HIC.

## Author contributions

CG-D, RR-L and PP-V contributed equally to this work in conception, design and writing of one of the three main sections of the manuscript, all together wrote the first draft of the manuscript and share the first authorship. L-VH, RC-C, MA-O, JP-C and AO-V contributed to the revision of the first draft of the manuscript. All authors contributed to the article and approved the submitted version.

## Conflict of interest

The authors declare that the research was conducted in the absence of any commercial or financial relationships that could be construed as a potential conflict of interest.

## Publisher’s note

All claims expressed in this article are solely those of the authors and do not necessarily represent those of their affiliated organizations, or those of the publisher, the editors and the reviewers. Any product that may be evaluated in this article, or claim that may be made by its manufacturer, is not guaranteed or endorsed by the publisher.
